# A metabolic reconstruction of *Lactobacillus reuteri* JCM 1112 and analysis of its potential as a cell factory

**DOI:** 10.1186/s12934-019-1229-3

**Published:** 2019-10-29

**Authors:** Thordis Kristjansdottir, Elleke F. Bosma, Filipe Branco dos Santos, Emre Özdemir, Markus J. Herrgård, Lucas França, Bruno Ferreira, Alex T. Nielsen, Steinn Gudmundsson

**Affiliations:** 1 Center for Systems Biology, School of Engineering and Natural Sciences, University of Iceland, Dunhagi 5, 107 Reykjavik, Iceland; 2 Matis, Vinlandsleid 12, 113 Reykjavik, Iceland; 3 The Novo Nordisk Foundation Center for Biosustainability, Technical University of Denmark, Building 220, Kemitorvet, 2800 Kgs. Lyngby, Denmark; 4 Molecular Microbial Physiology Group of the Swammerdam Institute for Life Sciences, University of Amsterdam, Science Park 904, 1098 XH Amsterdam, The Netherlands; 5 Biotrend SA – Biocant Park, Núcleo 04, Lote 2, 3060-197 Cantanhede, Portugal; 6 Present Address: Discovery, R&D, Chr. Hansen A/S, Bøge Allé 10-12, 2970 Hørsholm, Denmark

**Keywords:** *Lactobacillus reuteri*, Genome-scale metabolic model, Cell factory

## Abstract

**Background:**

*Lactobacillus reuteri* is a heterofermentative Lactic Acid Bacterium (LAB) that is commonly used for food fermentations and probiotic purposes. Due to its robust properties, it is also increasingly considered for use as a cell factory. It produces several industrially important compounds such as 1,3-propanediol and reuterin natively, but for cell factory purposes, developing improved strategies for engineering and fermentation optimization is crucial. Genome-scale metabolic models can be highly beneficial in guiding rational metabolic engineering. Reconstructing a reliable and a quantitatively accurate metabolic model requires extensive manual curation and incorporation of experimental data.

**Results:**

A genome-scale metabolic model of *L. reuteri* JCM 1112^T^ was reconstructed and the resulting model, Lreuteri_530, was validated and tested with experimental data. Several knowledge gaps in the metabolism were identified and resolved during this process, including presence/absence of glycolytic genes. Flux distribution between the two glycolytic pathways, the phosphoketolase and Embden–Meyerhof–Parnas pathways, varies considerably between LAB species and strains. As these pathways result in different energy yields, it is important to include strain-specific utilization of these pathways in the model. We determined experimentally that the Embden–Meyerhof–Parnas pathway carried at most 7% of the total glycolytic flux. Predicted growth rates from Lreuteri_530 were in good agreement with experimentally determined values. To further validate the prediction accuracy of Lreuteri_530, the predicted effects of glycerol addition and *adhE* gene knock-out, which results in impaired ethanol production, were compared to in vivo data. Examination of both growth rates and uptake- and secretion rates of the main metabolites in central metabolism demonstrated that the model was able to accurately predict the experimentally observed effects. Lastly, the potential of *L. reuteri* as a cell factory was investigated, resulting in a number of general metabolic engineering strategies.

**Conclusion:**

We have constructed a manually curated genome-scale metabolic model of *L. reuteri* JCM 1112^T^ that has been experimentally parameterized and validated and can accurately predict metabolic behavior of this important platform cell factory.

## Introduction

*Lactobacillus reuteri* is a heterofermentative Lactic Acid Bacterium (LAB) that is present in the human gut and is an important probiotic organism [52]. There is an increasing interest in using it as a cell factory for the production of green chemicals and fuels in a biorefinery [11, 44], due to its robustness properties. It has high growth and glycolytic rates, without the requirement for either aeration or strictly anaerobic conditions. It is tolerant to low pH, ethanol and salt, and has a wide growth temperature range. Moreover, it is genetically accessible, enabling metabolic engineering for cell factory optimization [3]. The species is known to produce 1,3-propanediol, reuterin, and other related industrially important compounds in high yields from glycerol [11], of which reuterin has also since long been known as antimicrobial [58]. *L. reuteri* also has most of the genes encoding for the enzymes needed for biosynthesis of 1,2-propanediol and 1-propanol, both of which are industrially relevant chemicals. These compounds are, however, not produced under normal conditions by *L. reuteri*, requiring improved engineering- and optimization strategies to achieve commercial level cell factories and production processes [7].

Genome-scale metabolic models are highly useful for directing metabolic engineering strategies, as well as to improve understanding of the physiology and metabolism of the target organism [43, 52]. So far, highly curated and experimentally validated metabolic models have been primarily developed for model organisms such as *Escherichia coli* and *Saccharomyces cerevisiae,* but models for several LAB species are also available, including *Lactobacillus plantarum* [61], *Lactobacillus casei* [62], *Lactococcus lactis* [16, 38] and *Streptococcus thermophilus* [42] (Table 1). These LAB are homofermentative or facultatively heterofermentative organisms and have substantial differences in metabolism compared to strict heterofermenters such as *L. reuteri* [3]. Metabolic models for the heterofermenters *Leuconostoc mesenteroides* [29, 39] are available (Table 1), but this is only distantly related to *L. reuteri* [3] and shows different metabolic features such as malolactic fermentation and a limited ability to use amino acids as energy source [29]. Models for two probiotic strains of *L. reuteri* have been previously published [52] (Table 1). They were automatically reconstructed from the same draft model we started with here [48]. The two previously published *L. reuteri* models were used along with transcriptomics data to identify qualitative metabolic differences between the two strains as well as to analyze their probiotic properties [52]. However, these previous models were not manually curated and were not used to quantitatively predict metabolic behavior. The construction of a genome-scale metabolic model that can be reliably used in basic research and cell factory design is a time-consuming process, requiring significant amount of manual curation and availability of strain-specific phenotypic data. At present, models obtained using automated tools or models that do not include experimental data are generally of limited use for quantitative predictions.

**Table 1:** LAB species with available genome-scale metabolic models

Species	Hetero-/homo-fermenter	Main applications and distinguishing characteristics	Genome size (Mb)	References
*Lactobacillus reuteri*	Hetero-	Produces Vitamin B12, 1,3-PDO, 3-HPA; used as probiotic and potential cell factory	2.0	[52], (This study)
*Leuconostoc mesenteroides*	Hetero-	Used in food fermentations (many non-dairy); malolactic fermentation; aroma production in foods	2.0	[29, 39]
*Lactobacillus plantarum*	Facultatively hetero-	Used in food fermentations, probiotics and potential cell factory	3.3	[61]
*Lactobacillus casei*	Facultatively hetero-	Used in food fermentations, probiotics and potential cell factory	2.9	[62]
*Lactococcus lactis*	Homo-	Used in dairy fermentations and potential cell factory	2.4	[38, 16]
*Streptococcus thermophilus*	Homo-	Used in dairy fermentations; fewer amino acid auxotrophies than other LAB, missing PPP genes	1.9	[42]

Here, we set out to reconstruct the metabolic network of *L. reuteri* JCM 1112, specifically for use in metabolic engineering applications, which requires collection of phenotypic data under several different conditions. We first performed an in-depth analysis of the genome to evaluate conflicting reports about metabolic pathways compared to strain DSM 20016. We then performed experiments to collect phenotypic data for the wild-type strain as well as for an alcohol dehydrogenase (*adhE)* knockout strain to constrain, validate, and test the model. Lastly, we use the model to test predictions for metabolic engineering strategies. The model as well as the experimental data are available in Additional files.

## Materials and methods

### Strains, media and culture conditions

Strains used in this study are listed in Table 2 and an overview of the experimental datasets in Table 3. All experiments were performed in triplicate except the one used for determining biomass composition and energy requirements as well as dataset B (Table 3). Apart from the growth mode, the dataset used for determining biomass and energy components and dataset A are identical, and the resulting data is in good agreement (Additional file 1). Dataset B was included, as it was available from the previous work that this paper builds upon [48], and is in good agreement with dataset D (Additional file 1).

**Table 2:** *Lactobacillus reuteri* strains used in this study

Strain name	Description/genotype	Origin/reference
JCM 1112 (DSM 20016, ‘WT’)	Wild-type	DSMZ^a^
SJ11774 (‘SJ (WT*)’)	Strain JCM 1112 (DSM 20016) with two inactivated restriction-modification systems (ΔLAR_RS04635 ΔLAR_RS07680::cat)	Novozymes; [7]
SJΔ*adhE*	Strain SJ11774 with a clean and full in-frame deletion of the bifunctional aldehyde/alcohol dehydrogenase *adhE* (LAR_RS01690)	Unpublished (manuscript in preparation)

^a^DSMZ = Deutsche Sammlung von Mikroorganismen und Zellkulturen

**Table 3:** Experimental datasets used for the model reconstruction

Strain	Substrate	Growth mode	Dataset name (Figures 4 + 5)	Used for:
WT	Glucose	Flask	–	Determining biomass composition and energy requirements (“Metabolic reconstruction” and “Resequencing reveals inconsistencies between the “same” strains *L. reuteri* DSM 20016 and JCM 1112—implications for glycolytic genes” sections)
WT	Glucose	Reactor	A	Model validation
WT	Glucose + glycerol	Reactor	B	Model validation
SJ (WT*)	Glucose	Flask	C	Model validation and model predictions
SJ (WT*)	Glucose + glycerol	Flask	D	Model validation and model predictions
SJΔadhE	Glucose	Flask	E	Model validation and model predictions
SJΔadhE	Glucose + glycerol	Flask	F	Model validation and model predictions

Growth curves and uptake and secretion data for all datasets can be found in Additional file 1

De Mann Rosa Sharp (MRS) medium (incl. 20 g/L glucose) was obtained from VWR and prepared according to the manufacturer’s instructions.

Chemically defined medium (CDM) was used as described in [48, 60] with the following modifications: arginine 5 g/L, tween-80 1 mL/L. Substrates were 111 mM glucose and 20 mM glycerol as indicated. The CDM was filter-sterilized and the final pH after mixing all components was 5.6.

All flask cultivations were performed in a stationary incubator at 37 °C. A 5 mm inoculation loop of culture was inoculated from − 80 °C glycerol stocks into 1 mL MRS with or without glycerol in a 1.5 mL Eppendorf tube and grown overnight (16 h). Next morning, cultures were washed 3× with sterile 0.9% NaCl, after which OD_600_ was measured and cells were transferred to 12 mL CDM with or without glycerol in a 15 mL Falcon tube to a starting OD_600_ of 0.08. After 4 h of growth, OD_600_ was measured and cultures were transferred to a starting OD_600_ of 0.05 in 100 mL pre-warmed CDM with or without glycerol in a 100 mL Schott flask. Samples for OD_600_ measurement and HPLC were taken directly after inoculation (t = 0 h) and at 2, 3, 4, 5, and 6 h; cultures were swirled for mixing prior to taking samples. The 6 h samples were also used for protein and amino acid determinations. The time points used were all during exponential growth, ensuring a pseudo steady state (Additional file 1).

All bioreactor cultivations were performed in batch mode and samples were taken during exponential/pseudo-steady state (Additional file 1). One of the fermentations was performed in CDM at 37 °C in 3.0 L bioreactors (BioFlo 115, New Brunswick Scientific/Eppendorf) with a 2.2 L working volume, 50 rpm agitation without gas sparging. The pH was controlled at 5.7 ± 0.1 using 5 N NaOH. Pre-cultures were performed similarly as for the flask cultures described above, with the pre-culture in CDM in 100 mL medium in 100 mL flasks, and reactors inoculated to an OD_600_ of 0.1. The other two reactor cultivations were performed in CDM, with and without glycerol, at 37 °C in 0.4 L reactors with a 0.5 L working volume, 50 rpm agitation and sparged with N_2_ at 15 mL/min for 1 h prior to inoculation. The pH was controlled at 5.8 using 5 M NaOH. Fermenters were inoculated to an initial OD_600_ of 0.05 from an exponentially growing culture on CDM without glycerol. As can be seen in Additional file 1, there is no difference between the cultures in the reactors that were sparged with N_2_ prior to fermentations and those that were not and hence we decided to treat these as replicates.

The correlation factor between cell dry weight (gDW) and OD_600_ was experimentally determined to be 0.4007 gDW/OD_600_ in CDM and used for calculating gDW from OD_600_ in all experiments.

### Analytical methods

Protein concentration of the cells was determined in the 6 h samples as described above, via a BCA protein assay (Merck-Millipore cat. 71285) according to the manufacturer’s protocol. Prior to the BCA assay, cell pellets were washed once in 0.9% NaCl and resuspended in 0.25 mM Tris–HCl pH 7.5 and sonicated on ice with an Ultrasonic Homogenizer 300VT (BioLogics) for 3 × 30 s at 40% power, with 30 s breaks on ice.

Amino acid composition of the cells was determined by Ansynth BV (The Netherlands) on washed cell pellets of a 6 h CDM culture as described above.

Substrates, products and amino acids secreted and taken up during the cultivations were quantified using HPLC. Glucose, glycerol, ethanol, lactate, acetate, citrate, 1,2-propanediol, 1,3-propanediol, 1-propanol, 2-propanol, pyruvate, succinate and malate were quantified with either one of two HPLCs: 1) a Dionex Ultimate 3000 (Thermo Scientific) containing an LPG-3400SD pump, a WPS-3000 autosampler, a UV–visible (UV–Vis) DAD-3000 detector, and an RI-101 refraction index detector. Injection volume was 20 µL. An Aminex HPx87 ion exclusion 125-0140 column was used with a mobile phase of 5 mM H_2_SO_4_, a flow rate of 0.6 mL/min and an oven temperature of 60 °C; 2) a Shimadzu LC-20AD equipped with refractive index and UV (210 nm) detectors, with an injection volume of 20 µL. A Shodex SH1011 8.0 mmIDx300mm column was used with a mobile phase of 5 mM H_2_SO_4_, a flow rate of 0.6 mL/min and an oven temperature of 50 °C. All amino acids, ornithine and GABA were quantified using a Dionex Ultimate 3000 (Thermo Scientific), for which the procedure is as follows: 20 µg/mL 2-aminobutanoic acid and sarcosine were used as internal standards for dilution of the samples; derivatization was performed in the autosampler. 0.5 µL sample was added into 2.5 µL of (v/v) 3-mercaptopropionic acid in borate buffer (0.4 M, pH 10.2), mixed and incubated for 20 s at 4 °C to reduce free cystines. Then 1 µL of 120 mM iodoacetic acid in 140 mM NaOH was added, mixed and incubated for 20 s at 4 °C to alkylate reduced cysteines. 1.5 µL of OPA reagent (10 mg *o*-phthalaldehyde/mL in 3-mercaptopropionic acid) was then added to derivatize primary amino acids. The reaction was mixed and incubated for 20 s at 4 °C. 1 µL of FMOC reagent (2.5 mg 9-fluorenylmethyl chloroformate/mL in acetonitrile) was added, mixed and incubated for 20 s at 4 °C to derivatize other amino acids. 50 µL of Buffer A (Buffer A: 40 mM Na_2_HPO_4_, 0.02% NaN_3_ (w/v) at pH 7.8) at pH 7 was added to lower the pH of the reaction prior to injecting the 56.5 µL reaction onto a Gemini C18 column (3 um, 4.6 × 150 mm, Phenomenex PN: 00F-4439-E0) with a guard column (SecurityGuard Gemini C18, Phenomenex PN: AJO-7597). The column temperature was kept at 37 °C in a thermostatic column compartment. The mobile phase had the following composition: Buffer A: see above, pH 7.8; Buffer B: 45% (v/v) acetonitrile, 45% (v/v) methanol and 10% (v/v) water; flow rate 1 mL/min. Derivatized amino acids were monitored using a fluorescence detector. OPA-derivatized amino acids were detected at 340_ex_ and 450_em_ nm and FMOC-derivatized amino acids at 266_ex_ and 305_em_ nm. Quantifications were based on standard curves derived from dilutions of a mixed amino acid standard (250 µg/mL). The upper and lower limits of quantification were 100 and 0.5 µg/mL, respectively.

### Genome sequencing and analysis

For genomic DNA (gDNA) isolation, overnight cultures of DSM 20016 and SJ 11774 were grown in MRS and the pellet was used for gDNA isolation using the Epicentre MasterPure™ Gram Positive DNA Purification kit according to the manufacturer’s protocol. Subsequent genome sequencing was performed at the sequencing facility at the NNF Center for Biosustainability. Library preparation was performed using KAPA HyperPlus Library Prep Kit (ROCHE) with Illumina-compatible dual-indexed PentAdapters (PentaBase). The average size of the library pool was 317 bp. Sequencing was performed on MiSeq (Illumina) using the MiSeq Reagent Kit v2, 300 Cycles (Illumina). The libraries were loaded to the flow cell at 10 pM and sequenced using paired-end reads of 150 bp. Read quality check was performed with FastQC version 0.11.5. Mutations relative to reference (*L. reuteri* JCM 1112, GenBank accession nr AP007281, annotated with Prokka version 1.11) were identified using Breseq (version 0.31.0) [10]. Mean coverage was 143.7x (SJ 11774) and 129.5x (DSM 20016). All runs were performed at the Danish national supercomputer for life sciences (Computerome), Technical University of Denmark. For this work, the annotated genome of *L. reuteri* JCM 1112 from NCBI was used. During the reconstruction, several genes were re-annotated, based on BLAST and physiological data. A list of all genes in the JCM 1112 genome can be found in Additional fie 2, along with annotations from the GenBank file and which model reactions are associated with each gene.

### Metabolic reconstruction

The *L. reuteri* JCM 1112 metabolic reconstruction was based on an unpublished, automatically generated draft reconstruction of JCM 1112 [48]. We performed extensive manual curation, including: gap filling, updating and adding gene-protein-reaction (GPR) associations, updating gene IDs, updating metabolite- and reaction abbreviations, in line with the BiGG database [28], updating and adding missing formulas and/or charges to metabolites, fixing unbalanced reactions, adding annotation to metabolites, reactions and genes and detailed review and integration of organism specific data. A biomass objective function was formulated based on available data on *L. reuteri* and related strains. The ATP cost of growth-associated maintenance (GAM) was estimated using one of the data sets (Table 3) by adjusting the GAM parameter so that growth predictions matched in vivo growth. This data set was then excluded from subsequent validation and prediction steps.

### Flux balance analysis

Flux balance analysis (FBA) was used to analyze the genome-scale metabolic model [15, 53] by constraining exchange reactions in the model with experimental values of substrate uptake and secretion rates. To take into account that the Embden–Meyerhof–Parnas (EMPP) pathway is a minor glycolytic pathway in *L. reuteri* compared to the phosphoketolase pathway (PKP) (“Curation process” section), an additional flux constraint was added to the model

$$ \frac{{v_{PFK} }}{{v_{PFK} + v_{G6PDH2r} }} \le r, $$

where *r* is an empirically determined flux ratio, v_PFK_ denotes flux in the rate limiting step of the EMPP and v_G6PDH2r_ is the flux in the first reaction branching into the PKP.We used a variant of FBA called parsimonious FBA [33] which identifies flux values corresponding to maximum growth with the side constraint that the sum of absolute flux values is made as small as possible. The sum of fluxes is proxy for enzyme usage and the method can therefore be considered to simulate biological pressure for rapid and efficient growth using minimum amount of resources (enzymes). An advantage over FBA is that the resulting solution is likely to contain fewer infeasible flux cycles. Model simulations were carried out in Python with the CobraPy toolbox [12] and GLPK solver. All code used in the simulations is provided in the form of a Jupyter notebook in Additional file 3 and on https://github.com/steinng/reuteri. The Escher package [27] was used for visualization of flux predictions. Escher maps of *L. reuteri*´s central metabolism are provided in Additional file 4, both simplified maps as shown in “Effects of adding glycerol and deleting *adhE*” and “Model-based analysis of 1-propanol production in *L. reuteri*” sections as well as a detailed map linking different sugar utilization pathways to the central metabolism.

To predict growth rates the model was constrained with uptake rates of glucose, glycerol and five amino acids (Arg, Ser, Asn, Asp and Glu), and with the secretion rates of ethanol, lactate, acetate and 1,3-propanediol. Effects of knocking out the *adhE* gene were predicted by temporarily deleting it from the network. Where the effects of an active 1,2-propanediol pathway were predicted, a methylglyoxal synthase (MGS) was added to the model and optimized for growth.

To predict the theoretical maximum yields of selected target compounds, a reaction enabling the secretion of the corresponding metabolite was added to the model, unless an exchange reaction already existed, and flux through the reaction maximized. The glucose uptake rate was 25.2 mmol/gDW/h, based on experimental data, and free secretion of by-products was allowed. For the production of l-alanine, an l-alanine dehydrogenase was added to the model. The production of ethyl lactate required the addition of a lactate acyl transferase and a reaction for the condensation of lactoyl-CoA with ethanol [32]. To produce 1-propanol, a methylglyoxal synthase (MGS) was added to the model. The presence of a complete 1-propanol pathway enables more efficient regeneration of NAD^+^ and the flux predictions were therefore repeated in the presence of an active MGS. To simulate a non-limiting phosphofructokinase, the flux constraint involving v_PFK_ above was omitted.

## Results and discussion

### Metabolic network reconstruction

To reconstruct a genome-scale metabolic model of *L. reuteri* suitable for use in cell factory design and optimization, we built upon a draft metabolic model of *L. reuteri* JCM 1112 described in [48] that we in turn extensively curated. The Memote tool [34] was used to assess the quality of the reconstruction and to guide the curation process (Additional file 5). The main characteristics of the resulting Lreuteri_530 model (Additional file 6) are listed in Table 4.

**Table 4:** Main characteristics of Lreuteri_530—the *L. reuteri* JCM 1112 genome-scale metabolic reconstruction

Genome characteristics	
Genome size	2.04 Mb
Total protein coding sequences	1943
Model characteristics	
Genes	530
Percentage of genome	27%
Reactions (with GPR)	710 (690)
Metabolites (unique)	658 (551)
Memote total score	62%

#### Curation process

Reactions and metabolites were abbreviated according to the BiGG database nomenclature where applicable and annotations with links to external databases included. Genes from the JCM 1112 genome were identified with locus tags from the GenBank file, and annotations were included which contain: the old locus tag which is often found in older literature, the NCBI protein ID, gene annotation and the protein sequence. Apart from general network curation, organism-specific information obtained from laboratory experiments and from available literature was integrated by reviewing reactions, genes and gene-protein-reaction (GPR) rules.

##### Resequencing reveals inconsistencies between the “same” strains *L. reuteri* DSM 20016 and JCM 1112—implications for glycolytic genes

The two most well-known strain names and origins for the type strain are DSM 20016 and JCM 1112 from the DSMZ and JCM culture collections, respectively. These two are derived from the same original human faeces isolate *L. reuteri* F275 [25], which was grown and stocked in two different laboratories [17]. Both genomes have been sequenced previously and a comparison showed that they are identical except for two regions that were missing in DSM 20016 [37], which were most likely lost during the 20 years of separate laboratory cultivation [17]. The first region (8435 bp, flanked by IS4 insertion sequences on each end) contains genes for glycolysis, namely glyceraldehyde-3-P dehydrogenase, phosphoglycerate kinase, triosephosphate isomerase, and enolase. The second region (30,237 bp, flanked by two different insertion sequence elements) contains for example a gene cluster for nitrate reductases and molybdopterin biosynthesis [37]. As the first island consists of glycolytic genes, the implications of its presence or absence are profound. This island is absent in DSM 20016, but we could identify homologs of all this island’s genes except glyceraldehyde-3-P dehydrogenase elsewhere in its genome based on annotation and/or BLAST.

During the preparation of our model, it became clear that there are inconsistencies in naming and hence gene content of the *L. reuteri* type strain. We sequenced the DSM 20016 strain that we obtained from DSMZ and this showed that its genome is identical to that of JCM 1112 instead, meaning it contained the two islands missing in DSM 20016. A similar result of these strains being ‘swapped’ was obtained by others based on whole genome sequencing [24] and PCR of part of the largest missing region in DSM 20016 in a study looking at cell-surface proteins in the different strains [13]. This inconsistency between the two strains does not seem to be commonly known and taken into account, and we suspect that some papers referring to either the DSM or the JCM strain might in fact be working with the other strain. For example, the DSM 20016 strain used by Sun et al. sequenced in 2015 (accession nr AZDD00000000), contains the islands as indicated by the presence of all glycolytic genes and hence is actually the JCM 1112 strain [56]. Contrarily, the DSM 20016 referred to by Morita et al. sequenced in 2007 by JGI (accession nr CP000705), was shown to be DSM 20016, missing the islands [37]. Both strains were obtained from DSMZ. This highlights the importance of re-sequencing of strains ordered from culture collections or lab strains present in the laboratory before using them for engineering or characterization studies. We strongly suggest that studies working with any *L. reuteri* type strain perform PCR on the two islands or perform resequencing to validate the presence or absence of the genes in the two islands.

Based on our sequencing results, we have included all genes in the two islands in our metabolic reconstruction and model. The genes in the model are identified with the locus tags obtained from the JCM 1112 strain’s genome in NCBI (NC_010609). As many other publications refer to genes in the DSM 20016 strain or use the old locus tags from the JCM 1112 genome, we have included a table (Additional file 2) which lists: the locus tags used in the model (gene numbers prefixed by LAR_RS), the old locus tags (gene numbers prefixed by LAR_), the annotations obtained from the NCBI GenBank file, the NCBI protein IDs (WP numbers), the locus tags of the corresponding genes in the DSM 20016 strain, when applicable (gene numbers prefixed by Lreu_), and finally the reaction(s) in the metabolic model associated with the genes.

##### Phosphofructokinase (PFK) and the distribution between EMP and PK pathway usage

**Fig. 1:**
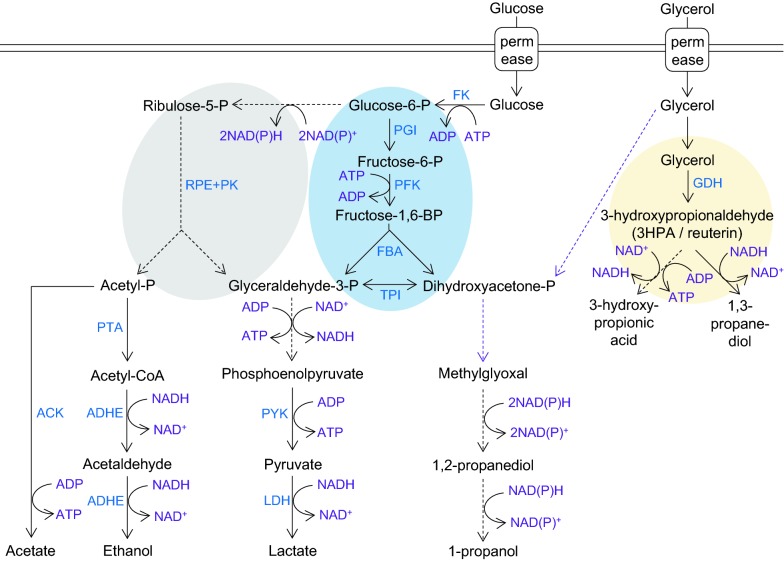
Condensed overview of the central metabolism in *L. reuteri*. Dotted purple arrows indicate pathways for which genes or homologs are present but likely not active in *L. reuteri* JCM 1112. Dotted black arrows indicate multiple enzymatic steps. Yellow background circle indicates microcompartment; blue background indicates the EMP pathway; grey background indicates the phosphoketolase pathway. FK: fructokinase/glucokinase; PGI: glucose-6-phosphate isomerase; PFK: phosphofructokinase; FBA: fructose-bis-phosphate aldolase; TPI: triosephosphate isomerase; PGM: phosphoglucomutase; RPE + PK: ribulose epimerase + phosphoketolase; GDH: glycerol dehydratase I; PTA: phosphotransacetylase; ACK: acetate kinase; ADHE: bifunctional aldehyde-alcohol dehydrogenase; PYK: pyruvate kinase; LDH: lactate dehydrogenase (Adapted from [3])

Obligately heterofermentative lactobacilli like *L. reuteri* are often considered to solely use the phosphoketolase pathway (PKP) instead of the Embden–Meyerhof–Parnas pathway (EMPP) for glucose consumption [3] (Fig. 1). Both pathways result in the glycolytic intermediate glyceraldehyde-3-phosphate but use different redox cofactors (Fig. 1). As the PKP yields one and the EMPP two molecules of glyceraldehyde-3-phosphate, the PKP has a lower energy yield than the EMPP (Fig. 1). The PKP generally results in the production of one molecule of lactate and one molecule of ethanol or acetate for one glucose molecule while the EMPP generally yields two lactate molecules. Key enzymes of the EMPP are fructokinase (FK), glucose-6-phosphate isomerase (PGI), phosphofructokinase (PFK), fructose-bis-phosphate aldolase (FBA), and triosephosphate isomerase (TPI). In line with the idea that heterofermenters use the PKP, Sun et al. showed in a comparison of 213 LAB genomes that *pfk* was lacking from a distinct monophyletic group formed by mainly (87%) obligatively and otherwise facultatively heterofermentative *Lactobacillus* spp., including *L. reuteri* DSM 20016 and *L. panis* DSM 6035 [56]. Contrary to most other species in the same group, these two species did contain *fba*, which has traditionally been linked to the presence of the EMPP. Despite the absence of *pfk*, EMPP activity has been observed in several *L. reuteri* strains and in some strains it appears to play a major role compared to the PKP, depending on the growth phase, and showing strain-specific differences [2, 4]. For modeling and engineering purposes, it is crucial to understand the presence and activity of the PKP vs the EMPP.

Årsköld et al. [2] compared the genomic organization of 13 sequenced *Lactobacillales* and showed that *L. reuteri* (strains ATCC 55730 and DSM 20016) is one of the four exceptions that do not have a *pfkA* gene where this is located in all other species. Nevertheless, they detect PFK and EMPP activity in strain ATCC 55730 and subsequently identify two genes (GenBank accession nrs EF547651 and EF547653) for orthologues of *pfkB*, a minor PFK-variant in *E. coli* [2]. In analogy with Årsköld et al. in *L. reuteri*, Kang et al. [26] identified a ribokinase in the obligately heterofermentative *L. panis* PM1 with 82% similarity to the *pfkB* gene identified in *L. reuteri* ATCC 55730 from Årsköld et al. (74% in our own BLAST search).

**Fig. 2:**
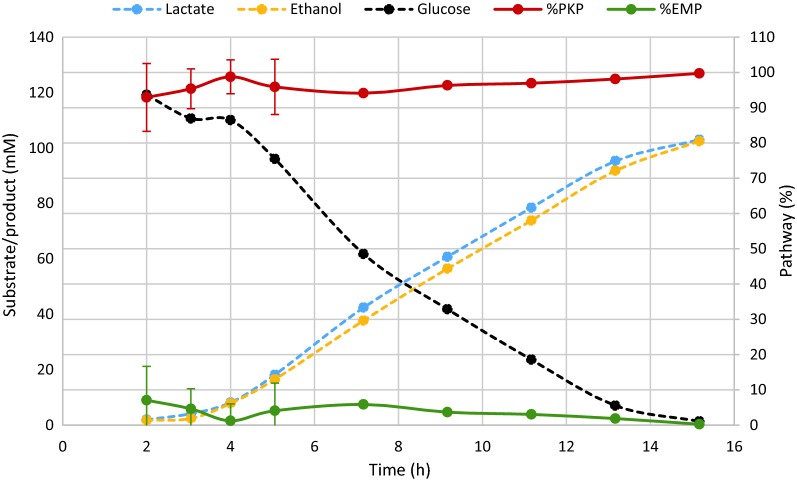
Typical fermentation profile and distribution between the EMP and PK pathways in *L. reuteri* JCM 1112 in chemically defined medium with glucose as the sole carbon source. Data are averages of the all the datasets used to constrain and validate the model, with error bars representing standard deviation. The percentage of PKP usage was defined as in Burgé et al. [4] i.e. as the ethanol concentration divided by the sum of lactate and ethanol concentrations divided by 2

A BLAST comparison of the *pfkB* protein sequence of *L. panis* PM1 (GenBank accession nr AGU90228.1) and *L. reuteri* ATCC 55730 (GenBank accession nr ABQ23677.1) against *L. reuteri* JCM 1112 resulted in 81% and 99% identity, respectively, to JCM 1112 gene number LAR_RS02150, which is annotated as ribokinase rbsK_2. On a gene level, this gene shares 97% identity with *L. reuteri* ATCC 55730 and 73% with *L. panis* PM1. The same identities were found in *L. reuteri* DSM 20016 for gene LREU_RS02105 (previously Lreu_0404, GenBank protein KRK49592.1). A second gene annotated as “ribokinase rbsK_3” (locus tag LAR_RS06895) showed only limited query coverage and identity and hence rbsK_2 is the most likely homolog of *pfkB*. The growth experiments conducted in the present study with JCM 1112 are in line with the findings of Burgé et al. [4] and indicate minor though detectable usage of the EMPP in this strain with a peak in the early growth stage (Fig. 2), in which this rbsK_2 likely fulfills the role of *pfkB*. The average flux through the EMPP in all cultures was 7.0% (Fig. 2) and was used to define the corresponding flux split ratio in the model (“Flux balance analysis” section).

##### Sugar transport

Transport of carbohydrates can be mediated by ATP-Binding Cassette (ABC) transporters, phosphotransferase systems (PTS), or secondary transporters (permeases of the Major Facilitator Superfamily, MFS) [47]. PTS systems mediate hexose mono- or dimer transport and phosphorylation simultaneously—mostly by using PEP to pyruvate conversion as phosphate donor, whereas ABC-transporters (mostly used for pentoses) and permeases (both pentoses and hexoses) perform only transport, and a separate ATP-utilizing kinase step is needed for sugar phosphorylation. Moreover, in Gram positives, PTS systems have an important role in carbon catabolite repression via phosphorylation cascades and direct interaction with the carbon catabolite repression protein A (ccpA) [18, 21]. Heterofermentative LAB contain fewer PTS system components than homofermentative LAB, which is thought to be the result of gene loss [67]. In general, organisms using the EMPP are believed to use PTS systems, and organism using the PKP to use secondary carriers [45]. Likely as a result of the lack of full PTS systems, glucose utilization is not constitutive but substrate-induced in heterofermenters, and utilization of several other sugars is not repressed by glucose [18]. Sugar transport in heterofermenters is poorly characterized, and only recently a study was dedicated to the genomic and phenotypic characterization of carbohydrate transport and metabolism in *L. reuteri*, as representative of heterofermentative LAB [66]. This showed that *L. reuteri* completely lacks PTS systems and ABC-transporters and solely relies on secondary transporters of the MFS superfamily, which use the proton motive force (PMF) as energy source for transport [66]. In *L. reuteri* JCM 1112, we could identify the two common proteins of the PTS system, Enzyme I (Lreu_1324) and HPr (Lreu_1325). Some sugar-specific parts were present, but no complete PTS was identified. As a result, all sugar transport in the model takes place via secondary transporters and the PMF.

##### Glycerol utilization

*Lactobacillus reuteri*, like many lactobacilli, is known to be unable to grow on glycerol as a sole carbon source, but can use it as an alternative electron acceptor, providing a means to gain energy on a variety of carbon sources [55, 57]. *L. reuteri* is among the best native producers of large amounts of 3-hydroxypropionaldehyde (reuterin, 3-HPA) from glycerol that are currently known [35]. This is an intermediate in the pathway to 1,3-propanediol (1,3-PDO, also produced by *L. reuteri*, depending on the conditions used) that is known to be toxic and produced in a microcompartment [5]. The reason why it cannot grow on glycerol as sole carbon source is currently not fully clear, although it is likely related to gene regulation. All the genes that are necessary to convert glycerol to dihydroxyacetone phosphate via either dihydroxyacetone (DHA) or glycerol-3-phosphate and hence shuttle it into glycolysis are present in the *L. reuteri* genome [5]. However, several of these genes have been shown to be downregulated in the presence of glycerol [5, 50]. Furthermore, the *L. reuteri* glycerol dehydrogenase also has activity as 1,3-PDO:NAD-oxidoreductase, whereas in for example *Klebsiella pneumoniae*, which does produce glycolytic end products from glycerol, these are two different enzymes [57]. It seems that the physiological role of this enzyme in *L. reuteri* is the reduction of 3-HPA to 1,3-PDO, rather than glycerol to DHA conversion, explaining the lack of growth on glycerol [57].

##### Other pathways

Most heterofermentative LAB possess a malolactic enzyme but no malic enzymes [31], which is also the case for our *L. reuteri* strain, based on sequence comparisons with the *L. casei* strain used by Landete et al. [31]. Based on BLAST analysis and in line with literature, *L. reuteri* JCM 1112 possesses a malate dehydrogenase and PEP carboxykinase, and cannot utilize citrate; malate (and fumarate) is converted to succinate [20].

From a biotechnological perspective, an interesting branch point of central carbon metabolism is the conversion from methylglyoxal (MG) to 1,2-propanediol (1,2-PDO), which can then be further metabolized into 1-propanol and propanoate. *L. reuteri* possesses all enzymes needed for these pathways, except methylglyoxal synthase (MGS), the step of the pathway converting dihydroxyacetone phosphate into MG [19, 55]. It has been shown that when MG is added to *L. reuteri* JCM 1112 cultures or when a heterologous *mgs* is expressed, all the subsequent metabolites are formed [7]. Although we identified a potential distant homolog of *mgs* in the *L. reuteri* genome, this homolog is clearly not active under normal conditions since no 1,2-PDO was observed in our experiments. Hence, all the genes in these pathways except *mgs* were included in the reconstruction. For methylglyoxal reductase, *mgr*, we also identified several aldo/keto reductases as possible homologs, based BLAST comparison to genes identified in [19]. However, verification of these hypothetical activities would need extensive enzyme assays, and it is also likely that this reaction is performed by LAR_RS09730 (Glycerol dehydrogenase) [1, 65], which has been added to the reconstruction for the MGR reaction. Alternatively, MG might be converted directly to lactate by a glyoxalase  [19].

Whereas many LAB are auxotrophic for vitamin B12, *L. reuteri* is a native producer. Vitamin B12 is important as a cofactor in for example the 3-HPA pathway but is also of relevance for biotechnological and medical/health applications (e.g. when produced by probiotic strains). The structure and biosynthetic genes have been studied in detail [49, 50]. The corresponding pathway is present in the reconstruction and is active during growth predictions.

#### Biomass reaction and energy requirements

A biomass objective function (BOF), which contains all necessary components for biomass biosynthesis, is commonly used to predict growth rate in metabolic models. Ideally, the BOF should be constructed based on organism-specific experimental data, mainly the fractional composition of the macromolecules (proteins, DNA, RNA, lipids, etc.) and their individual building blocks (amino acids, nucleotides, fatty acids, etc.), as well as the energy necessary for their biosynthesis [14]. The protein fraction is a significant fraction of the biomass and was therefore measured experimentally. The ratio of individual amino acids in the *L. reuteri* biomass was also measured experimentally. The remaining macromolecular fractions were derived from *L. plantarum* [61] and *L. lactis* [38]. Nucleotide composition was estimated from the genome, which in the case of RNA is not ideal since it assumes equal transcription of all genes. We however preferred to use this approximation instead of using experimental data from another organism. Fatty acid composition of *L. reuteri* was obtained from literature [36], while phospholipid composition was adopted from *L. plantarum*. The composition of lipoteichoic acid [63] and exopolysaccharides [30] in *L. reuteri* were obtained from literature. Peptidoglycan composition was adopted from *L. plantarum* and glycogen was assumed to be negligible [8, 9].

Energy required for growth (GAM) and cell maintenance (NGAM) are important parameters in metabolic models, and can be estimated from ATP production rates, which can be calculated from experimental data obtained at different dilution rates [59]. Unfortunately, this data is not publicly available for *L. reuteri*. These parameters have been estimated from experimental data for several other LAB, including *L. plantarum*, and reported in literature [61]. Even though *L. reuteri* and *L. plantarum* are relatively closely related, adopting these parameters from *L. plantarum* can negatively affect the quality of model predictions. When the differences in physiologies of *L. plantarum* and *L. reuteri* are considered, it is possible that *L. reuteri* requires less energy: (1) The genome is only ~ 2 Mb, while *L. plantarum*’s genome is 3.3 Mb. (2) *L. reuteri* is an obligate heterofermenter, which means it uses almost solely the PKP (Fig. 2) to break down glucose, resulting in one ATP per glucose, while a facultative heterofermenter like *L. plantarum* uses the EMPP when grown on glucose, resulting in two ATPs. (3) LAB in general have low catabolic capabilities, and for *L. reuteri* this includes auxotrophy for several amino acids. This, combined with the fact that macromolecular biosynthesis is already accounted for in the model reactions, supports the claim that adopting energy parameters from* L. plantarum* can negatively affect model predictions, as we also observed when evaluating this in our model. We decided to use one of our experimental datasets (Table 3) to estimate the GAM value, while using the NGAM value from *L. plantarum* (“Metabolic reconstruction” section). In general, NGAM represents only a small portion of the total energy requirements of the cell and therefore has much smaller effect on model predictions than GAM. This resulted in a GAM value of 10.2 mmol/gDW/h. Detailed description of the biomass reaction, relevant data and calculations can be found in Additional file 7.

The sensitivity of the predicted growth rate to changes in biomass and energy components was investigated by varying the coefficient of each component, one at a time, by 50% while varying the glucose uptake rate. The components tested were protein, polysaccharide, DNA, RNA, lipid, GAM and NGAM. The analysis showed that predicted growth rate was sensitive to changes in the protein and GAM components of the biomass, compared to the other components (see figure in Additional file 7). As described earlier, these two particular components of the biomass are based on* L. reuteri* specific experimental data obtained in this study.

#### Lactobacillus reuteri model compared to models of* L. lactis* and* L. plantarum*

**Fig. 3:**
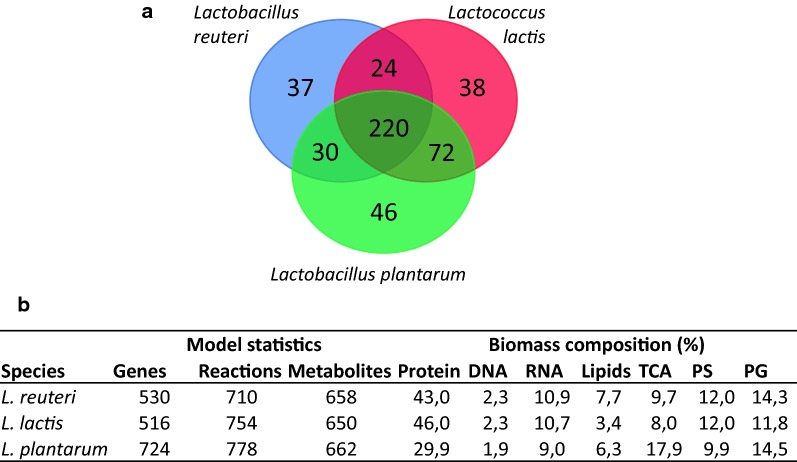
Comparison of genome-scale metabolic models of *L. reuteri*, *L. lactis* and *L. plantarum*.** a** Venn diagram showing number of EC-numbers that represent common and unique reactions in the three models.** b** Model statistics for the three strains, along with biomass ratio

The model was compared to genome-scale metabolic models of two other LAB, *L. lactis* and *L. plantarum* (Table 1). Common and unique metabolic reactions were analyzed based on EC-numbers (Fig. 3a). Unique reactions in *L. reuteri* included reactions belonging to: cofactor and prosthetic group biosynthesis, most of which related to B-12 vitamin synthesis; alternative carbon metabolism, such as glycerol; amino acid metabolism, which can be explained by the different amino acid auxotrophies among the three strains; methylglyoxal metabolism (see Additional file 8 for more details). Basic model statistics and biomass composition from the three models are presented in Fig. 3b. Comparing biomass ratios shows that *L. plantarum*´s biomass contains less protein than the others and more teichoic acid. Model predictions reflected the well-established and previously discussed differences in glycolytic pathways between the strains, namely how *L. lactis* and *L. plantarum*, as homofermenter and facultative heterofermenters, use the EMP pathway resulting in higher energy compared to* L. reuteri* which, as a strict heterofermenters, mostly uses the PKP.

### Model applications

#### Model validation using experimental data: Growth rate comparisons

**Fig. 4:**
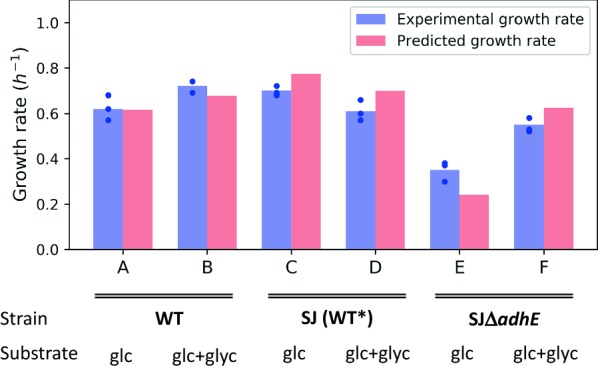
Predicted and experimental growth rates. Experimentally measured growth rates for each of the six data sets are shown in blue, with blue dots denoting individual replicates and blue bars representing average values. For each dataset, the model was constrained with average experimental values for uptake and secretion rates of carbon sources, byproducts and selected amino acids, and optimized for growth. Predicted growth rates are represented by red bars. Different datasets used are indicated with letters—glc: glucose; glyc: glycerol

To validate the model, several different datasets (Table 3) with measured uptake- and secretion rates of carbon sources, amino acids and organic byproducts were used to constrain exchange fluxes in the model. The predicted growth rates were compared with observed experimental growth rates (Fig. 4). In all cases, flux through the EMPP was set to maximally 7% based on the experimentally determined value (Fig. 1). The chemically defined culture medium used in the growth experiments contained all 20 amino acids, except for l-glutamine. Subsequently, all these amino acids were quantified during growth and the model was constrained with the resulting uptake rates. Of all the amino acids, only arginine was depleted at the end of the exponential phases in data sets A, B and C (Additional file 1). Due to auxotrophy for several amino acids (Glu, His, Thr, Arg, Tyr, Val, Met, Try, Phe, Leu), the model is highly sensitive to uncertainties in measurements, as well as in determined protein- and amino acid fractions of the biomass reaction. To accurately represent amino acids in the biomass reaction, both the protein content and the amino acid ratio were measured (Additional file 7). By enabling unrestricted uptake of amino acids in the model, we noticed that only 5 amino acids (Arg, Ser, Asn, Asp, Glu) needed to be constrained with measured uptake rates for accurate growth predictions, for both the wild-type and the mutant. This is due to their role in energy- and cofactor metabolism, not only in biomass biosynthesis. Hence, only this minimum number of amino acids was used to constrain the model in the following. The remainder were assumed to be non-limiting by allowing unrestricted uptake. This has twofold advantage. First, it limits the effects of uncertainties in amino acid uptake rate measurements on model predictions, a problem exacerbated by the amino acid auxotrophy. Second, it simplifies future applications of the model by reducing the number of measurements needed.

In most cases, model predictions and in vivo data were in good agreement (Fig. 4). Datasets C and D in Fig. 4 show a variant of the WT strain (marked SJ (WT*)), which lacks two restriction modification (RM) systems for easier genetic manipulation (Table 2). Datasets E and F show a mutant derived of the SJ strain with a clean and in-frame deletion of the *adhE* gene (bifunctional aldehyde/alcohol dehydrogenase). The model predicts slightly higher growth rates than observed in vivo for the SJ strain (datasets C and D in Fig. 4) and the mutant strain grown on glucose and glycerol (F in Fig. 4). Unexpectedly, the RM-modifications in the SJ strain seem to slightly alter its behavior on CDM with glucose and glycerol compared to the WT (Additional file 1). For the mutant strain grown on glucose (dataset E in Fig. 4), the model predicts a slightly lower growth rate than observed in vivo, though both show a large decrease in growth, compared to the WT. The most likely explanation for this is that some glucose is being taken up in vivo, even though the measurements did not show this (the likely amount consumed between two samples is within the error of the assay). Secretion of 2.6 mmol/gDW/h of lactate and 2.7 mmol/gDW/h of acetate was observed in vivo. The model, however, does not predict lactate and acetate secretion unless some glucose uptake is allowed. If a glucose uptake of 2.6 mmol/gDW/h is allowed, the growth rate increases from 0.22 to 0.34 h^−1^, compared to 0.30 h^−1^ in vivo. Amino acid measurements showed that the mutant in dataset E used l-arginine to a greater extent than the WT, which the model predicts is used to generate energy via the arginine deiminase pathway, resulting in increased growth.

#### Effects of adding glycerol and deleting adhE

**Fig. 5:**
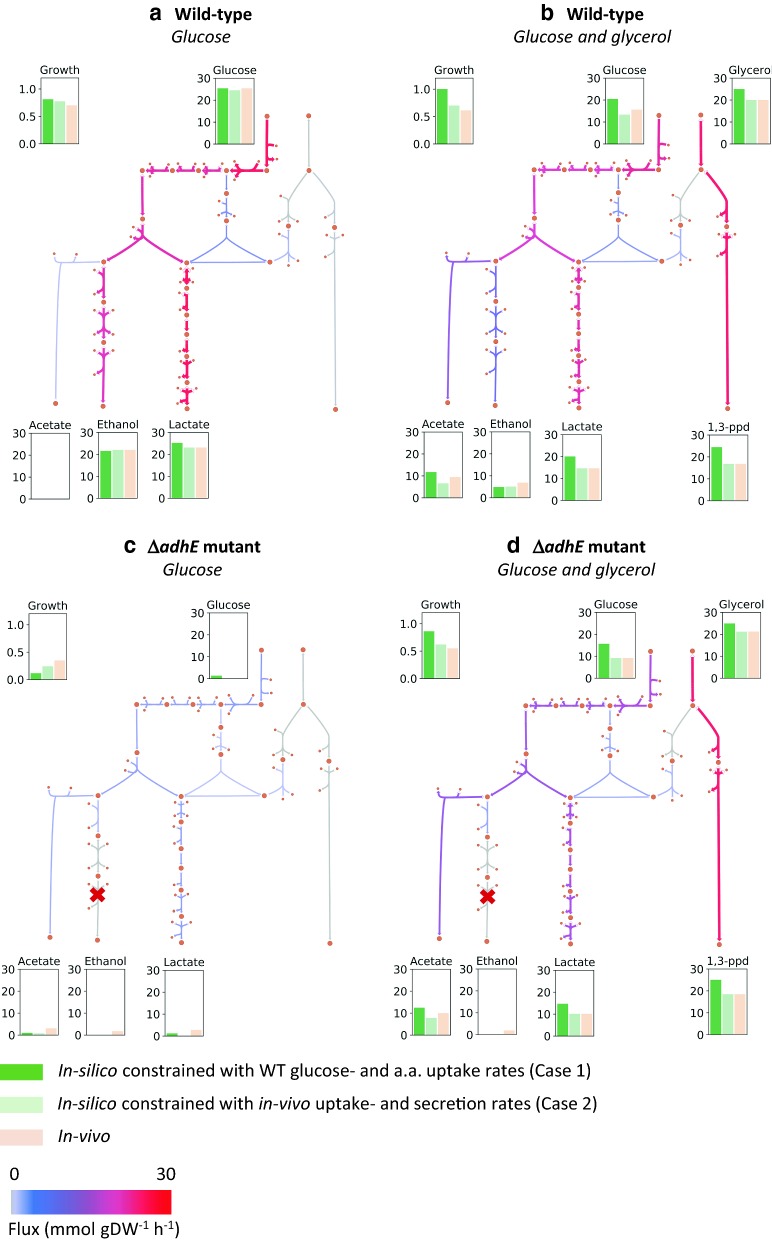
Predicted and experimental fluxes of key metabolites in the wild-type strain (SJ) and the *adhE* mutant. The wild-type strain was grown on glucose (**a**) and glucose and glycerol (**b**), and the *adhE* mutant was also grown on glucose (**c**) and glucose and glycerol (**d**). Bar plots show the average measured rates from 3 replicates (light orange), predicted rates from model constrained with average experimental uptake rates of the WT grown on glucose, or case 1 (dark green), and predicted rates from model constrained with average experimental rates from the strain and condition under study, or case 2 (light green). Metabolic maps show predicted flux distributions for case 1. All units for uptake- and secretion rates are in mmol gDW^−1^ h^−1^ and for growth rates in h^−1^. Metabolic maps correspond to Fig. 1. Amino acids (a.a.) in case 1 refer to Arg, Ser, Asn, Asp and Glu

To investigate the applicability of the model for cell factory design, it was used to predict the effects of adding glycerol to the glucose-based culture medium, as well as knocking out the *adhE* gene, which plays a critical role in ethanol production and redox balance (Fig. 1). The datasets used here are the same as in the previous section (datasets C–F in Fig. 4). There, the aim was to validate the model by means of comparing predicted growth rates to experimentally determined growth rates. In this section, we look more specifically at predicted flux distributions in central metabolism, both with and without strain- and condition-specific experimentally determined constraints. For this purpose, we studied two cases in order to answer the following questions: (1) If the model is constrained only with experimentally determined glucose- and five amino acid uptake rates from the WT strain grown on glucose, how do the predicted effects of glycerol addition and/or *adhE* knock-out (dark green bars in Fig. 5) compare to in vivo growth rate and uptake- and secretion measurements (light orange bars in Fig. 5)? This was tested to evaluate the applicability of the model in a practical setting. One of the main goals of using a model like this should be to probe the effects of genetic and media perturbations in silico, i.e. without having to do extensive condition-specific cultivations and measurements beforehand. (2) If the model is constrained with uptake- and secretion rates of carbon source(s), amino acids and byproducts of the strain and condition under study, how well do the model predictions (light green bars in Fig. 5) compare to in vivo results? Here the model was allowed, but not forced, to take up (lower bound constrained, upper bound unconstrained) and secrete (lower bound unconstrained, upper bound constrained) metabolites according to the experimental data. This tells us if the model, when imposed with realistic limitations, “chooses” a flux distribution which results in extracellular fluxes of metabolites in line with in vivo data. In both cases, the constrained amino acids only included Arg, Ser, Asn, Asp and Glu as before (“Model validation using experimental data: growth rate comparisons” section) and in case 1 the allowed glycerol uptake rate was arbitrarily limited to 25 mmol/gDW/h, when glycerol effects were being predicted.

The flux maps in Fig. 5 show results for case 1 (dark green bars). The predicted uptake of glucose and glycerol (dark green bars in Fig. 5b) is higher than observed in vivo (light orange bars in Fig. 5b), resulting in higher secretion of by-products and a higher growth rate as well. However, the distribution of secreted by-products is very similar. The effect of glycerol can be predicted quite well with the model as ethanol secretion decreases and acetate secretion increases, relative to glucose uptake, and 1,3-propanediol is secreted in large amounts (compared to graphs in Fig. 5a). Several studies have described an increased growth rate in *L. reuteri* when glycerol is added to a glucose-based medium (in flasks and bioreactors), which is to be expected based on inspection of redox balance [5, 48, 57] and this is also what we observed in silico in case 1. *L. reuteri* uses practically only the PKP and not the EMP for glucose fermentation. In the PKP, two extra NAD(P)H molecules are formed compared to the EMP, which are regenerated to NAD(P)^+^ by AdhE through the formation of ethanol (Fig. 1). When glycerol is added, it is used as an alternative electron acceptor via the production of 1,3-PDO, which generates one NAD^+^. As a result, one of the actetyl-phosphates that is normally converted to ethanol can now be converted to acetate. This does not yield NAD^+^ (which is now regenerated in 1,3-PDO production) but does yield one ATP, enabling a higher growth rate [5, 48]. Along these lines of reasoning and in line with existing literature [5], knocking out the *adhE* gene has dramatic effects on the metabolism when glucose is the sole carbon source, both in vivo and in silico (Fig. 5c). When ethanol production was inactive, the growth decreased which also led to reduction in lactate production. This is due to redox imbalance since AdhE no longer recycles the NADH generated in glycolysis. The predictions in case 1 show highly decreased uptake of glucose, yet a small amount of glucose is still taken up, resulting in acetate and lactate production. As discussed in Model validation using experimental data: Growth rate comparisons, it is possible that glucose is being taken up in vivo, even though this is not detected by measurements, which is in line with model predictions and would also explain the lower growth rate observed in silico in case 2 compared to in vivo. The higher growth rate in vivo compared to in silico in case 1 is due to a much higher arginine uptake than measured in the WT. Also in line with published studies and the redox balance explained above [5], addition of glycerol to the *adhE* mutant increases the growth rate to almost WT levels (Fig. 5d). Similarly to the WT predictions, the model in case 1 predicts slightly higher growth rate and uptake rates of glucose and glycerol, resulting in higher secretion of by-products. But as before, the flux distribution is very similar to the one measured in vivo.

In all four conditions in Fig. 5 the in silico predictions in case 2 and the in vivo data are almost identical, with the exception of the few instances described above. In few cases discrepancies can be explained by carbon imbalance in vivo, which is most likely due to measurement uncertainties. Taken together, these results show that the model can be used to accurately predict metabolic behavior, without requiring extensive experimental data.

#### Model-based analysis of 1-propanol production in *L. reuteri*

**Fig. 6:**
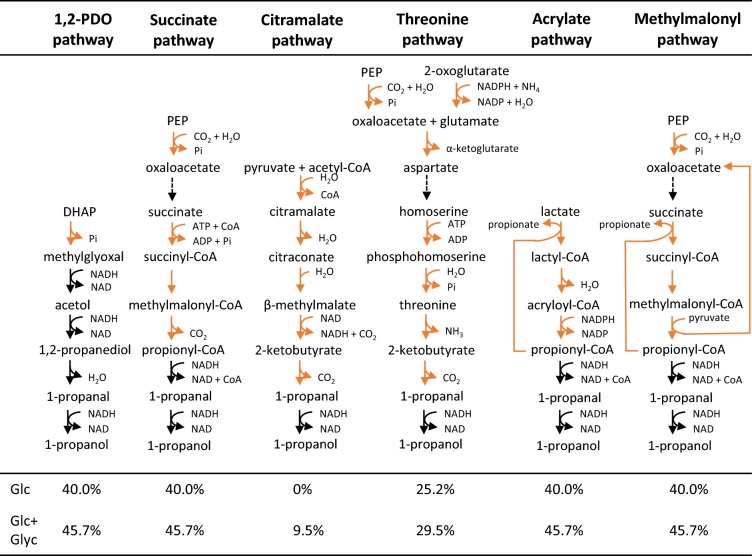
1-propanol biosynthetic pathways tested in silico. Black reactions are natively present and yellow are heterologous. Theoretical carbon yields, obtained by maximizing for 1-propanol production on either glucose or glucose and glycerol, are listed under each pathway

In the two previous sections we used experimental data to validate model predictions. In this section we focus on in silico predictions involving the production of 1-propanol. It has been shown that heterologous expression of methylglyoxal synthase (*mgs*) in *L. reuteri* can activate the pathway to 1,2-PDO and 1-propanol production [7]. Both these compounds have many applications, e.g. in the production of polyester resins for 1,2-PDO and as a solvent and potential biofuel for 1-propanol [23]. In addition to the 1,2-PDO pathway, several different pathways have been described for 1-propanol production [64]. The 1,2-PDO pathway towards 1-propanol has not frequently been reported for 1-propanol production; the most frequently used pathways are the citramalate and threonine pathways (Fig. 6). Other options are the acetone, Wood-Werkman (or methylmalonyl), acrylate and succinate pathways [64]. For a recent extensive overview of these pathways and engineered and non-engineered organisms, the reader is referred to the review by Walther and Francois [64]. The thermodynamic maximum yield of 1-propanol from glucose calculated based on the degree of reduction is 1.33 mol/mol (or 44,4% carbon yield). However, only the stoichiometry of the 1,2-PDO, succinate, acrylate and Wood-Werkman pathways allow this maximum yield—the others result in up to 25% less yield [64]. Here, we evaluated the citramalate, threonine, succinate, acrylate, methylmalonyl, and 1,2-PDO pathway. One of the advantages of *L. reuteri* for using the 1,2-PDO pathway towards 1-propanol is that *L. reuteri* produces vitamin B12, which is needed as a co-factor for the B12-dependent diol dehydratase step and has been suggested to be a limiting factor in this pathway in for example *C. glutamicum* [54] and was added to the fermentations in an* E. coli* strain harboring this pathway [22]. The model was used to analyze the suitability of six different pathways for 1-propanol production in* L. reuteri* (Fig. 6). These were the known pathways as described in literature, but in addition we used the minRxn algorithm [6] and the accompanying database of enzymatic reactions to search for heterologous pathways from glucose to 1-propanol. This search did not reveal pathways that differed significantly from the already-known pathways as shown in Fig. 6, neither qualitatively nor in terms of carbon yields (data not shown). The model was maximized for 1-propanol production, while constrained with the experimentally determined uptake rate of glucose and additional 20 mmol/gDW/h of glycerol when applicable, no uptake of amino acids and free secretion of by-products. Of the six pathways in Fig. 6, the citramalate pathway performed the worst, with no production on glucose and only 9.5% maximum carbon yield when glycerol was added. This was due to redox imbalance, which was due to lack of production of the precursors pyruvate and acetyl-CoA, and was partly fixed by adding glycerol. Adding this pathway in combination with the threonine pathway did not increase maximum carbon yield compared to threonine pathway alone. The highest carbon yields, 40.0% and 45.7% on glucose and glucose + glycerol, respectively, were observed in four different pathways, namely the 1,2-PDO, succinate, acrylate and methylmalonyl pathways. Since the maximum theoretical carbon yield of this pathway is 44.4% on glucose and 50.8% on glucose + glycerol, these values represent around 90% of the theoretical maximum, which can be achieved with minimal metabolic engineering (i.e. only heterologous expression of mgs). Since for the 1,2-PDO pathway only one heterologous enzyme is needed, we decided to analyze 1-propanol production via the 1,2-PDO pathway further.

**Fig. 7:**
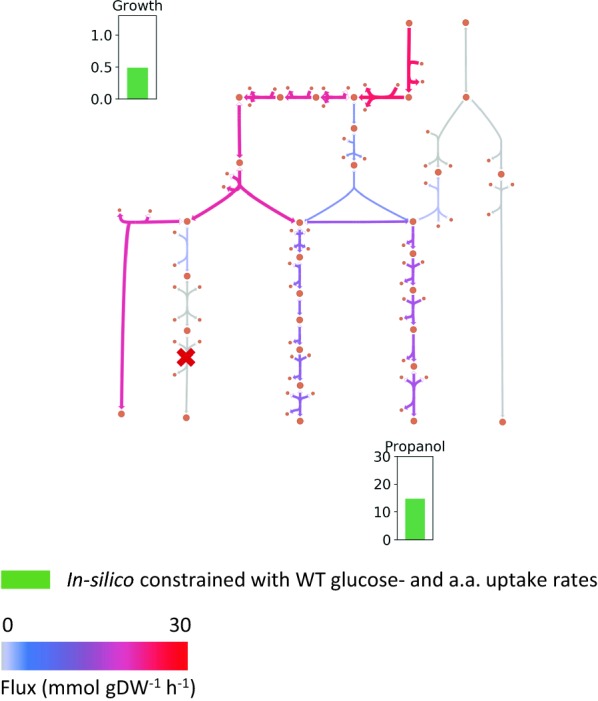
Predicted flux distribution, growth rate and 1-propanol production of *adhE* mutant grown on glucose, with active 1,2-propanediol and 1-propanol pathways. The model was constrained with average experimental uptake rates of the WT grown on glucose and optimized for growth. Units for propanol secretion rate is in mmol gDW^−1^ h^−1^ and growth rate in h^−1^. Amico acids (a.a.) refer to Arg, Ser, Asn, Asp and Glu

While optimizing in silico for 1-propanol production as described above is a good way to assess suitability of different engineering strategies, optimizing for growth gives more biologically realistic results. Hence, next the model was used to maximize growth while constrained with experimental uptake rates of glucose and the 5 amino acids from the WT grown on glucose (same as case 1 in “Effects of adding glycerol and deleting adhE section”). As previously discussed (“Effects of adding glycerol and deleting adhE section”), the *adhE* mutant grows poorly on glucose due to redox imbalance. The synthesis of both 1,2-propanediol and 1-propanol consumes NADH and activating these pathways therefore has the potential to restore growth. The *adhE* gene was knocked out in silico, and flux predictions with (Fig. 7) and without (Fig. 5c) an active 1,2-PDO pathway were compared. The active 1,2-PDO pathway resulted in a high increase in growth rate (0.11 to 0.49 h^−1^) as well as growth-coupled production of 1-propanol (14.7 mmol/gDW/h). Given the good agreement between in silico predictions and in vivo measurements in “Effects of adding glycerol and deleting adhE section”, the expression of the missing *mgs* gene in the 1,2-PDO pathway at a sufficiently high level in vivo is expected to result in a relatively fast-growing 1-propanol-producing cell factory, which is also in agreement with existing literature [7].

#### Model-based analysis of *L. reuteri* as a cell factory

LAB are natural producers of several chemicals of industrial interest [3, 40, 51]. They possess high sugar uptake rates and, in many species, the central metabolism is only weakly coupled to biomass formation because of their adaptation to nutrient rich environments. As a result, the carbon source is mostly used for energy gain and is converted to fermentation products in high yields. Combined with high tolerance to environmental stress, these properties have led to significant interest in using LAB as cell factories.

The heterofermentative nature of *L. reuteri* and the dominance of the phosphoketolase over the Embden–Meyerhof–Parnas pathway make some target compounds less suitable than others, with lactic acid being an obvious example. On the other hand, these properties can also be used to an advantage as is demonstrated here. We used our newly established *L. reuteri* metabolic model to study the feasibility of this organism to produce some of the compounds that have been the subject of recently published LAB metabolic engineering experiments. These native and non-native compounds include a flavoring compound (acetoin), a food additive (l-alanine), biofuels (1-propanol and ethanol), chemical building blocks (acetaldehyde and 2,3-butanediol) and an environmentally friendly solvent (ethyl lactate). The last compound has recently been produced in an engineered* E. coli* strain [32] and is an interesting target in *L. reuteri* since it is a condensation product of the two major products of glucose fermentation via the phosphoketolase pathway, lactate and ethanol.

The suitability of *L. reuteri* for producing a particular compound was assessed in terms of the maximum carbon yield, using a fixed glucose uptake rate (Table 5). This gives an overly optimistic estimate of product yields in most cases since it completely ignores variations in enzyme efficiency, compound toxicity, regulation and other issues outside the scope of the model. The maximum flux is still useful to identify products that appear to be ill suited for a particular metabolism as well as products that may be suitable.

**Table 5:** Model predictions of the maximum theoretical yields of selected target compounds

Compound	Maximum flux (mmol gDW^−1^ h^−1^)			Maximum carbon yield (%)
		MGS	MGS, ↑-PFK	
Ethanol	50.4	50.4	50.4	67
Acetaldehyde	0	31.5	37.8	50
1-Propanol (n–n)	20.2	20.2	20.2	40
l-Alanine (n–n)	27.0	27.0	50.4	100
Acetoin	0	10.1	18.9	50
2,3-Butanediol	0	11.6	21.6	57
Ethyl lactate (n–n)	20.4	20.4	25.2	83

A maximum glucose uptake rate of 25.2 mmol gDW^−1^ h^−1^ was assumed. MGS indicates the presence of methylglyoxal synthase in the model, ↑-PFK indicates the presence of a phosphofructokinase that is not flux-limiting. Non-native compounds are indicated with (n–n)

The predicted flux for acetaldehyde, acetoin and 2,3-butanediol, which are all derived from acetyl-CoA, was low, suggesting that the metabolism in the wild type is not well suited for overproducing these compounds. The flux increased significantly upon addition of methylglyoxal synthase, suggesting the importance of the 1-propanol pathway in cofactor balancing (“Model-based analysis of 1-propanol production in *L. reuteri*” section). Addition of glycerol to the medium served the same purpose and increased the predicted flux in all cases (data not shown), which is in line with glycerol being known and used as an external electron sink in *L. reuteri* [11]. For all the compounds except ethanol and 1-propanol, the addition of a fully functional phosphofructokinase was predicted to increase the yields even further (Table 5). Such a strategy has been shown successful for mannitol production [41].

Taken together, the model suggests that *L. reuteri* is better suited for producing compounds derived from pyruvate than compounds derived from acetyl-CoA and that the simultaneous expression of heterologous MGS and PFK enzymes is a general metabolic engineering strategy for increasing product yields in *L. reuteri*.

## Conclusions

In this study, we have established a manually curated genome-scale metabolic model of *L. reuteri* JCM 1112, referred to as Lreuteri_530, and validated it with experimental data. We identified several knowledge gaps in the metabolism of this organism that we resolved with a combination of experimentation and modeling. The distribution of flux between the PKP and EMPP pathways is strain-specific and in line with other studies, we found that the EMPP activity is maximally around 7% of total glycolytic flux during early exponential phase. The predictive accuracy of the model was estimated by comparing predictions with experimental data. Several scenarios were tested both in vivo and in silico, including addition of glycerol to a glucose-based growth medium and the deletion of the *adhE* gene, which encodes a bifunctional aldehyde/alcohol dehydrogenase. The results showed that the model gives accurate predictions, both with respect to growth rate and uptake- and secretion rates of main metabolites in the central metabolism. This indicates that the model can be useful for predicting metabolic engineering strategies, such as growth-coupled production of 1-propanol. The model also serves as a starting point for the modeling of other *L. reuteri* strains and related species. The model is available in SBML, Matlab and JSON formats at https://github.com/steinng/reuteri as well as in Additional file 6. Metabolic maps in Escher format are provided in Additional file 4. The Escher maps together with the model in JSON format can be used directly with the Escher-FBA online tool [46] as well as the Caffeine cell factory design and analysis platform (https://caffeine.dd-decaf.eu/).

## Supplementary information

Additional file 1. Growth curves and measured uptake- and secretion rates of metabolites for experimental datasets.

Additional file 2. Gene list for *L. reuteri* JCM 1112.

Additional file 3. Code used for simulations.

Additional file 4. Escher metabolic maps.

Additional file 5. Memote report.

Additional file 6. Genome scale metabolic model: Lreuteri_530.

Additional file 7. Biomass reaction.

Additional file 8. Unique EC-numbers for *L. reuteri*, *L. plantarum* and *L. lactis*.

## Data Availability

The model, experimental data, code and other relevant material are available from github.com/steinng/reuteri and additional files.
