# Supplementary material for: A metabolic reconstruction of Lactobacillus reuteri JCM 1112 and analysis of its potential as a cell factory
Source: Microb Cell Fact. 2019 Oct 29;18:186. doi: 10.1186/s12934-019-1229-3 (PMC6821008; doi:10.1186/s12934-019-1229-3)
Supplement: Supplementary file 6 — Additional file 6. Genome scale metabolic model: Lreuteri_530. [file 12934_2019_1229_MOESM6_ESM.html]

MemoteReportApp
