# Supplementary material for: A metabolic reconstruction of Lactobacillus reuteri JCM 1112 and analysis of its potential as a cell factory
Source: Microb Cell Fact. 2019 Oct 29;18:186. doi: 10.1186/s12934-019-1229-3 (PMC6821008; doi:10.1186/s12934-019-1229-3)
Supplement: Supplementary file 7 — Additional file 7. Biomass reaction. [file 12934_2019_1229_MOESM7_ESM.pdf]

Biomass composition of *L. reuteri*

Protein:DNA:RNA:Lipid:LTA:CPS:PG ratio of biomass is not known for reuteri. Lipoteichoic acid (LTA) from *L. lactis* (Oliveira, Nielsen, & Förster, 2005) is used because *L. plantarum* also produces Wall teichoic acid (WTA), which *L. reuteri* and *L. lactis* do not. Peptidoglycan in *L. plantarum* (Teusink et al., 2006) was calculated from *Bacillus cereus*, so here peptidoglycan from *L. lactis* is used. Protein ratio was experimentally measured for *L. reuteri* in this study. Otherwise numbers from *L. plantarum* were used for the macromolecular composition.

**Component**		**Fraction (% w/w)**	**Norm Fraction (% w/w)**	**Molar mass (g mol^{-1})**	**Coefficent (mmol gDW^{-1})**	**Source**
Protein	PROT_LRE_c	35,4%	43,0%	129,9	3,311	*L. reuteri* (This study)
DNA	DNA_LRE_c	1,9%	2,3%	309,5	0,075	*L. plantarum* (Teusink et al., 2006)
RNA	RNA_LRE_c	9,0%	10,9%	321,6	0,340	*L. plantarum* (Teusink et al., 2006)
Lipids	LIP_LRE_c	6,3%	7,7%	795,1	0,096	*L. plantarum* (Teusink et al., 2006)
Lipoteichoic acid	LTAtotal_LRE_c	8,0%	9,7%	5290,4	0,018	*L. lactis* (Oliveira et al., 2005)
Polysaccharides	CPS_LRE_c	9,9%	12,0%	648	0,186	*L. plantarum* (Teusink et al., 2006)
Peptidoglycan	PGlac2_c	11,8%	14,3%	992	0,145	*L. lactis* (Oliveira et al., 2005)
	Total	82,3%	100%			

0.186 CPS_LRE_c + 0.075 DNA_LRE_c + 0.096 LIP_LRE_c + 0.018 LTAtotal_LRE_c + 0.145 PGlac2_c + 3.311 PROT_LRE_c + 0.340 RNA_LRE_c + 10.2 atp_c + 1e-05 btn_c + 0.0002 coa_c + 10.2 h2o_c + 0.0002 nad_c + 1e-06 pydx5p_c + 1e-05 thf_c + 1e-05 thmpp_c + 0.0002 udcpdp_c + 0.00001 adeadocbl_c --> 10.2 adp_c + 10.2 h_c + 10.2 pi_c

Flux through ATPM is fixed at: 0.36 mmol h^{-1} gDW^{-1} (*L. plantarum* (Teusink et al., 2006))

Protein

Amino acid composition analyzed following cell pellet hydrolysis (Ansynth).

**Amino Acid**	**Fraction g/g$_{\text{total a.a.}}$**	**Fraction g/gDW**	**Fraction g/gDW (only protein)**	**MW (g/mol)**	**Fraction mole/gDW**	**Molar ratio**
Ala	10,4%	5,2%	0,2%	89,1	2,25E-05	0,7%*
Arg	4,7%	2,4%	2,4%	174,2	1,35E-04	4,1%
Asp + Asn	13,4%	6,7%	6,7%	132,1	5,08E-04	
Asp						7,7%
Asn						7,7%
Cys	0,8%	0,4%	0,4%	121,2	3,15E-05	1,0%
Glu + Gln	13,8%	6,9%	4,8%	146,6	3,27E-04	
Glu						2,7%*

Gln						7,1%
Gly	5,2%	2,6%	2,6%	75,1	3,50E-04	10,6%
His	2,1%	1,1%	1,1%	155,2	6,93E-05	2,1%
Ile	4,9%	2,5%	2,5%	131,1	1,89E-04	5,7%
Leu	7,3%	3,7%	3,7%	131,1	2,81E-04	8,5%
Lys	9,4%	4,7%	4,7%	146,2	3,23E-04	9,8%
Met	2,7%	1,3%	1,3%	149,2	8,93E-05	2,7%
Phe	3,5%	1,8%	1,8%	165,2	1,08E-04	3,3%
Pro	3,0%	1,5%	1,5%	115,1	1,33E-04	4,0%
Ser	3,8%	1,9%	1,9%	105,1	1,80E-04	5,4%
Thr	5,1%	2,5%	2,5%	119,1	2,13E-04	6,4%
Trp	0,9%	0,5%	0,5%	204,2	2,31E-05	0,7%
Tyr	3,4%	1,7%	1,7%	181,2	9,30E-05	2,8%
Val	5,4%	2,7%	2,7%	117,2	2,33E-04	7,0%
**Total**	**100%**	**50,2%**	**43,0%**	**129,9**	**3,31E-03**	**100%**

*Glutamate and alanine in lipoteichoic acid and peptidoglycan are subtracted from measured values.

Alanine in lipoteichoic acid:

mmol LTA / gDW	0,018
mol alanine / mol LTA	15,3
mol alanine in LTA / gDW	0,0003
Alanine molar mass (g/mol)	89,094
**g alanine in LTA / gDW**	**0,0245**

Alanine in peptidoglycan:

mmol PGlac2 / gDW	0,145
mol alanine / mol LTA	2
mol alanine in LTA / gDW	0,0003
Alanine molar mass (g/mol)	89,094
**g alanine in LTA / gDW**	**0,0258**

Glutamate in Peptidoglycan:

mmol PGlac2 / gDW	0,145
mol glutamate / mol LTA	1
mol glutamate in LTA / gDW	0,0002
Glutamate molar mass (g/mol)	147,13
**g glutamate in LTA / gDW**	**0,0213**

Protein reaction:
0.306 atp_c + 2 gtp_c + 2.306 h2o_c + 0.0062 alatrna_c + 0.0412 argtrna_c + 0.0775 asntrna_c + 0.0775 asptrna_c + 0.0096 cystrna_c + 0.0721 glntrna_c + 0.0189 glutrna_c + 0.1068 glytrna_c + 0.0211 histrna_c + 0.0578 iletrna_c + 0.0857 leutrna_c + 0.0986 lystrna_c + 0.0272 mettrna_c + 0.0329 phetrna_c + 0.0404 protrna_c + 0.0548 sertrna_c + 0.0650 thrtrna_c + 0.0070 trptrna_c +

0.0284 tyrtrna_c + 0.0711 valtrna_c --> PROT_LRE_c + 0.306 adp_c + 2.0 gdp_c + 2.306 h_c + 2.306 pi_c + 0.0062 trnaala_c + 0.0412 trnaarg_c + 0.0775 trnaasn_c + 0.0775 trnaasp_c + 0.0096 trnacys_c + 0.0910 trnaglu_c + 0.1068 trnagly_c + 0.0211 trnahis_c + 0.0578 trnaile_c + 0.0857 trnaleu_c + 0.0986 trnalys_c + 0.0272 trnamet_c + 0.0329 trnaphe_c + 0.0404 trnapro_c + 0.0548 trnaser_c + 0.0650 trnathr_c + 0.0070 trnatrp_c + 0.0284 trnatyr_c + 0.0711 trnaval_c

DNA

Calculated from JCM1112 genome

Nucleotide	Ratio	MW (g mol^{-1})	MW * Ratio (g mol^{-1})
dAMP	0,30560617	331,2	101,2
dTMP	0,30560617	304,2	93,0
dCMP	0,19439383	289,2	56,2
dGMP	0,19439383	304,2	59,1
		**Average MW of DNA**	**309,5**

DNA reaction:
1.37 atp_c + 0.310284595735 datp_c + 0.180906354753 dctp_c + 0.207484087701 dgtp_c + 0.301324961811 dttp_c + 1.37 h2o_c --> DNA_LRE_c + 1.37 adp_c + 1.37 h_c + 1.37 pi_c + ppi_c

RNA

Calculated from JCM1112 genome, assuming equal transcription

Nucleotide	Ratio	MW (g mol^{-1})	MW * Ratio (g mol^{-1})
AMP	0,31434679	329,2	103,5
UMP	0,29106444	306,2	89,1
CMP	0,18074957	305,2	55,2
GMP	0,21383921	345,2	73,8
		**Average MW of RNA**	**321,6**

RNA reaction:
0.710810743235 atp_c + 0.187747387186 ctp_c + 0.21245220899 gtp_c + 0.288989660589 utp_c --> RNA_LRE_c + 0.4 adp_c + 0.4 h_c + 0.4 pi_c + ppi_c

Lipids (Liu, Hou, Zhang, Zeng, & Qiao, 2014)

Fatty acid	Ratio	MW (g mol^{-1})	Formula	MW * Ratio (g mol^{-1})
C14:0	0,0687	228,38	$C_{14}H_{28}O_2$	15,68971
C16:0	0,2566	256,43	$C_{16}H_{32}O_2$	65,79994
C16:1	0,0258	254,41	$C_{16}H_{30}O_2$	6,563778
C18:0	0,075	284,48	$C_{18}H_{36}O_2$	21,336
C18:1	0,1591	282,47	$C_{18}H_{34}O_2$	44,94098
C18:2	0,3519	280,45	$C_{18}H_{32}O_2$	98,69036
C18:3	0,0418	278,44	$C_{18}H_{30}O_2$	11,63879
cyc19:0	0,0211	296,49	$C_{19}H_{36}O_2$	6,255939
			**Average MW of fatty acids**	**270,9155**

Fatty acids:

agly3p_LRE_c + 0.0211 cpocdacp_c + 0.0258 hdeACP_c + 0.075 ocdacp_c + 0.0418 ocdctrACP_c + 0.3519 ocdcyaACP_c + 0.1591 octeACP_c + 0.2566 palmACP_c + 0.0687 tdeacp_c --> ACP_c + pa_LRE_c

Phospholipids ratio from *L. plantarum* used here, as no *L. reuteri* data was available

Phospholipid	Ratio	MW with average fatty acid (g mol^{-1})	MW * Ratio (g mol^{-1})
Phosphatidylglycerol	0,75	751,95	563,96
1-lysyl phosphatidylglycerol	0,23	882,14	202,89
Cardiolipin	0,02	1411,81	28,24
**Average MW of phospholipids**			**795,1**

Lipid reaction:
0.02 clpn_LRE_c + 0.23 lyspg_LRE_c + 0.75 pg_LRE_c --> LIP_LRE_c

Lipoteichoic acid (Bron, Baarlen, & Kleerebezem, 2011; Kleerebezem et al., 2010; Walter et al., 2007)

20 glycerol phosphate (Gro-P) residues

Gro-P residues were substituted with d-alanyl esters (74-79%, average 76,5%)

Gro-P residues were substituted with glycosyl residues (6%)

Component	Average molar ratio	Molar mass (g mol^{-1})
Diglucosyl diacylglycerol	1	906,3
Glycerol phosphate	20	154,0
D-alanine	15,3	71,1
D-glucose	1,2	180,2
**Average molar mass of Lipoteichoic acid**		**5290,4**

LTA reaction:
0.175 LTA_LRE_c + 0.765 LTAala_LRE_c + 0.06 LTAglc_LRE_c --> LTAtotal_LRE_c

L. reuteri does not produce Wall teichoic acid

Polysaccharides (Ksonzeková et al., 2016)

Glucan homopolysaccharides (merely glucose units)

D-glucose, 4 units, 162 g mol^{-1} per unit → 648 g mol^{-1}

Polysaccharide reaction:
4.0 h2o_c + 4.0 udpg_c <=> CPS_LRE_c + 5.0 h_c + 3.0 udp_c + ump_c

Peptidoglycan

Assumed the same as *L. plantarum*

Peptidoglycan reaction:
uaagmdalac_c --> PGlac2_c + udcpdp_c

Glycogen

Glycogen in *B. subtilis* biomass was negligible (Dauner & Sauer, 2001; Dauner, Storni, & Sauer, 2001). Assumed the same here.

ATP requirements

No data for *L. reuteri* available. Non-growth associated maintenance (NGAM) assumed the same as *L. plantarum.* Growth associated maintenance (GAM) was calculated by constraining model with experimental uptake- and secretion rates of metabolites, and growth rate of *L. reuteri*.

Sensitivity analysis

The effect of 50% variation in biomass and energy component coefficients on predicted growth rate was investigated. Growth rate was most sensitive to change in the protein and GAM components of the biomass.

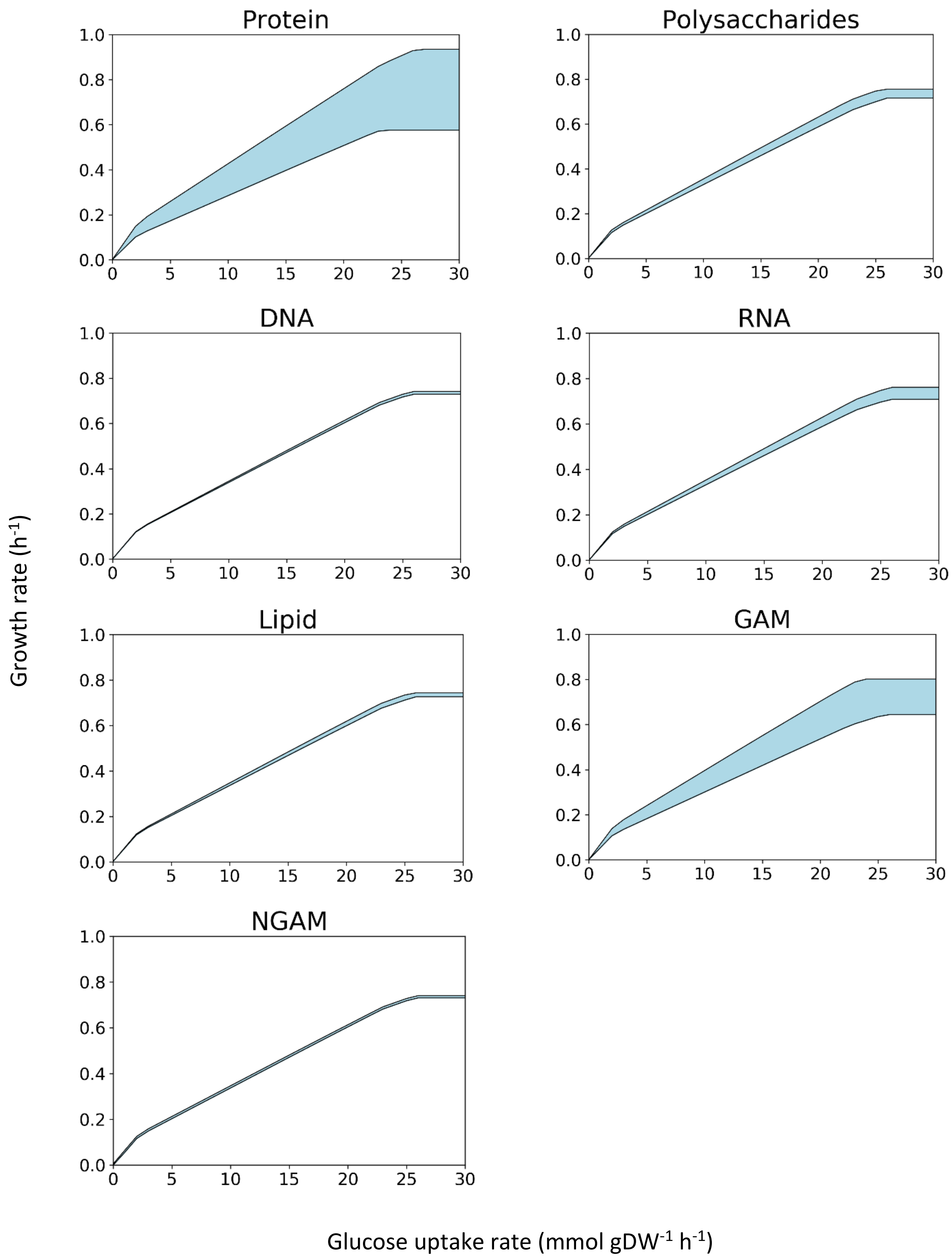

References

Bron, P. A., Baarlen, P. Van, & Kleerebezem, M. (2011). Emerging molecular insights into the interaction between probiotics and the host intestinal mucosa. *Nature Reviews Microbiology*, *10*(1), 66–78. https://doi.org/10.1038/nrmicro2690

Dauner, M., & Sauer, U. (2001). Stoichiometric Growth Model for Riboflavin-Producing Bacillus subtilis. *Biotechnology and Bioengineering*, *76*(2), 132–143.

Dauner, M., Storni, T., & Sauer, U. W. E. (2001). Bacillus subtilis Metabolism and Energetics in Carbon-Limited and Excess-Carbon Chemostat Culture. *Journal of Bacteriology*, *183*(24), 7308–7317. https://doi.org/10.1128/JB.183.24.7308

Kleerebezem, M., Hols, P., Bernard, E., Rolain, T., Zhou, M., Roland, J., & Bron, P. A. (2010). The extracellular biology of the lactobacilli. *FEMS Microbiology Reveiws*, *34*, 199–230. https://doi.org/10.1111/j.1574-6976.2009.00208.x

Ksonzeková, P., Bystricky, P., Vlcková, S., Pätoprst, V., Pulzová, L., Mudronová, D., … Tkáciková, L. (2016). Exopolysaccharides of Lactobacillus reuteri : Their influence on adherence of E . coli to epithelial cells and inflammatory response. *Carbohydrate Polymers*, *141*, 10–19. https://doi.org/10.1016/j.carbpol.2015.12.037

Liu, X. T., Hou, C. L., Zhang, J., Zeng, X. F., & Qiao, S. Y. (2014). Fermentation conditions influence the fatty acid composition of the membranes of Lactobacillus reuteri I5007 and its survival following freeze-drying. *Letters in Applied Microbiology*, *59*, 398–403. https://doi.org/10.1111/lam.12292

Oliveira, A. P., Nielsen, J., & Förster, J. (2005). Modeling Lactococcus lactis using a genome-scale flux model. *BMC Microbiology*, *5*(1). https://doi.org/10.1186/1471-2180-5-39

Teusink, B., Wiersma, A., Molenaar, D., Francke, C., De Vos, W. M., Siezen, R. J., & Smid, E. J. (2006). Analysis of growth of Lactobacillus plantarum WCFS1 on a complex medium using a genome-scale metabolic model. *Journal of Biological Chemistry*, *281*(52), 40041–40048. https://doi.org/10.1074/jbc.M606263200

Walter, J., Loach, D. M., Alqumber, M., Rockel, C., Hermann, C., Pfitzenmaier, M., & Tannock, G. W. (2007). D-Alanyl ester depletion of teichoic acids in Lactobacillus reuteri 100-23 results in impaired colonization of the mouse gastrointestinal tract. *Environmental Microbiology*, *9*(7), 1750–1760. https://doi.org/10.1111/j.1462-2920.2007.01292.x
